# Determinants of equivalent pathways and substitution effects on adolescents’ physical activity within family contexts: a configurational analysis based on FsQCA method

**DOI:** 10.1186/s12889-026-26327-2

**Published:** 2026-01-23

**Authors:** Xianwei Zhou, Xinying Zhang, Zhiqing Yang, Yuanyuan Ma, Yiyi Chen, Kefeng Li

**Affiliations:** 1 School of Physical Education, University of Jinan, Jinan, China; 2 School of Law, Shanxi University of Finance and Economics, Taiyuan, China; 3 School of Physical Science, Jishou University, Jishou, China; 4 School of Physical Education, Jinan Preschool Education College, Jinan, China

**Keywords:** Adolescent physical activity, Family determinants, Equivalent pathways, Substitution effects, FsQCA

## Abstract

**Background:**

Lack of physical activity among adolescents has become one of the biggest public health issues facing many countries. The family, being the primary environment for adolescents, directly impacts their level of physical activity. This study aims to reveal equivalent pathways and substitution effects of family determinants that effectively drive adolescent physical activity levels.

**Methods:**

This study employs fuzzy-set qualitative comparative analysis (fsQCA) to conduct a stratified sampling survey of 1,738 students from 12 middle schools in the eastern, central, and western regions of China. Research data were collected through standardized questionnaires covering demographic characteristics, Physical Activity Rating Scale (PARS-3), Chinese version of the Short Form of the Parental Bonding Instrument (S-EMBU-C), Parent-Child Relationship Scale (PRC), and Family Sports Atmosphere Questionnaire (FSAQ). The study focuses on how family structure factors (family economic status, family structure) and process-oriented factors (parenting styles, parent-child relationships, family sports atmosphere) form equivalent paths and substitution effects through synergistic mechanisms, thereby enabling adolescents to meet the standard of 60 min of moderate-to-vigorous physical activity (MVPA) daily.

**Results:**

A single factor is not a necessary condition for motivating adolescents to participate in physical activities; there are four equivalent pathways to motivate adolescents to engage in physical activities: the first is Family Economic Status (FES) * ~Family Structure (FS) * Parent-Child Relationship (PR) * Family Sports Atmosphere (FSA); the second is Family Economic Status (FES) * Positive Parenting Style (PPS); the third is Family Economic Status (FES) * ~Positive Parenting Style (PPS) * Parent-Child Relationship (PR) * ~Family Sports Atmosphere (FSA); the fourth is ~ Family Economic Status (FES) * Family Structure (FS) * Positive Parenting Style (PPS) * ~Family Sports Atmosphere (FSA). There exists substitution effects among structural factors, process factors, and between them, meaning that when one or more structural factors or process factors are absent, other structural factors, process factors, or combinations of factors can drive adolescents’ physical activity behaviors.

**Conclusion:**

The synergistic effects of structural and process factors within the family environment are more effective in driving adolescents’ participation in physical activities. Therefore, implementing family intervention measures requires simultaneous optimization of family resource allocation and interaction mechanisms, which provides a certain scientific basis for constructing a multidimensional collaborative family health promotion system.

## Introduction

Insufficient physical activity (PA) has been confirmed as the fourth leading independent risk factor for global mortality [1]. Moderate-to-vigorous physical activity (MVPA) effectively promotes cardiovascular and respiratory function, musculoskeletal development in adolescents, and reduces the risk of chronic non-communicable diseases such as obesity and type 2 diabetes [2]. However, epidemiological surveys in China reveal that children and adolescents average only 37.66 min of MVPA per day, which is 42.34% short of the recommended 60 min according to the Chinese Guidelines for Physical Activity in Children and Adolescents. Additionally, this level of activity decreases with age, dropping by 28.7% from elementary to middle school [3, 4].

Social cognitive theory reveals that individual health behaviors are influenced by the mutual interaction of personal traits, environmental factors, and the behavior itself [5]. As the core micro-environment for adolescent growth, the family plays an irreplaceable role in shaping their health behaviors [6], especially during the period of shrinking family size in China (the average family size in China was only 2.62 people in 2020) [7]. The impact of family factors on adolescents’ physical activity is multidimensional and complex. For instance, family economic capital provides foundational support through resource supply, while a family sports atmosphere shapes behavioral templates through intergenerational transmission. Parenting styles directly mediate the quality of participation in physical activities [8–11]. Additionally, adolescents from reconstituted families and single-parent families have lower levels of physical activity engagement and are associated with higher psychological stress and health management risks [12–15]. Structural variables in the family environment, such as economic status and family type, often exert their effects through process mechanisms like parent-child interactions and parenting styles, making it inappropriate to draw conclusions solely based on structural variables [16–19].

However, existing studies focus more on the linear relationship between antecedent variables and adolescents’ physical activity [14–17], while the combined effects and substitution relationships of these antecedent variables have not been sufficiently explained. Given this, this study integrates key variables such as family type, economic status, sports atmosphere, parenting style, and parent-child relationships, aiming to introduce fuzzy set qualitative comparative analysis (fsQCA) to reveal the synergistic effects from a configurational perspective. This approach seeks to identify equivalent paths and substitution effects of different variable combinations, providing a reference for implementing intervention strategies at the family level.

## Methods

### Study participants

**Fig. 1:**
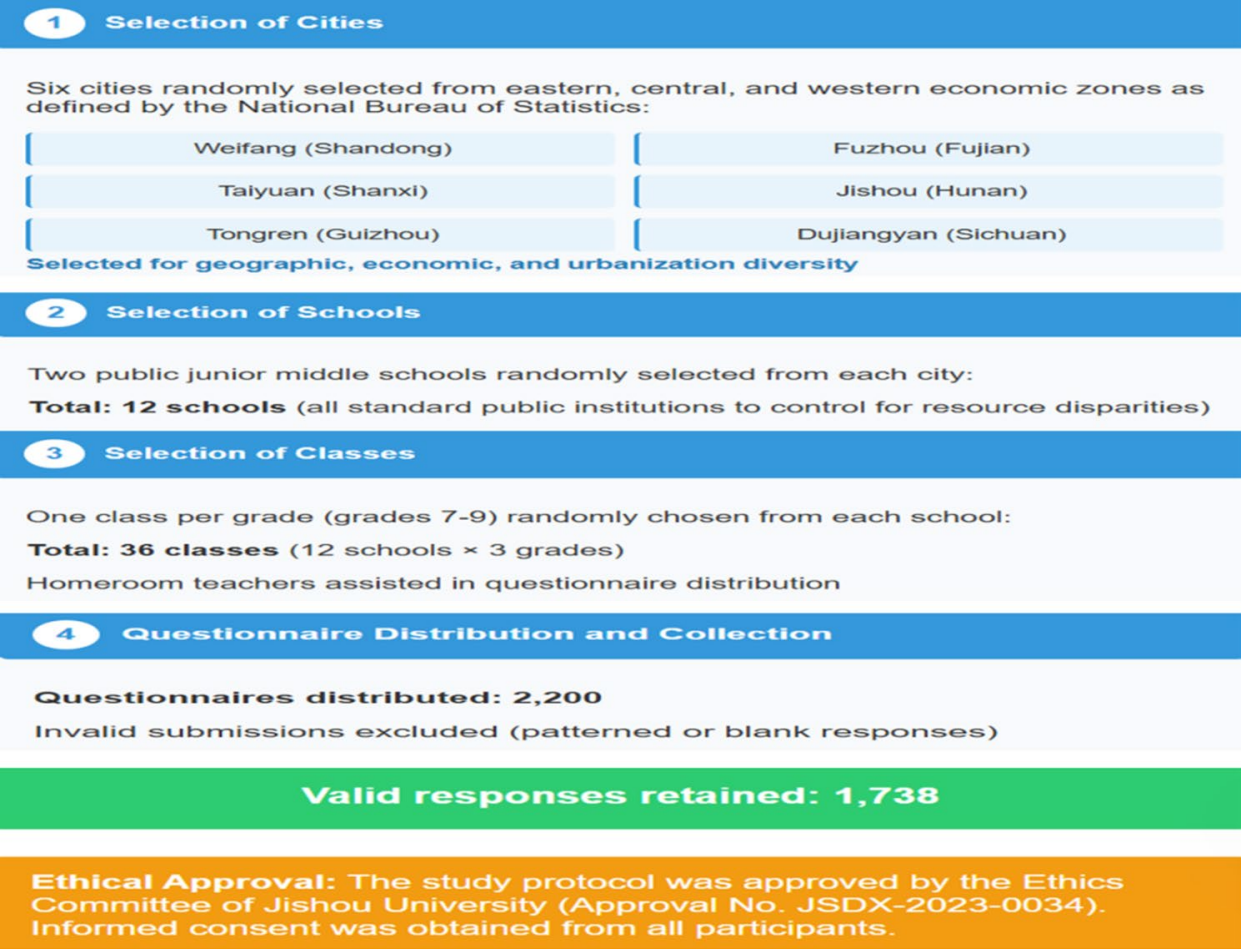
Flow chart for inclusion of participants

To ensure the sample represented regions with varying socioeconomic levels in China, this study employed a multi-stage random cluster sampling method in November and December 2023. Initially, six cities—Weifang (Shandong), Fuzhou (Fujian), Taiyuan (Shanxi), Jishou (Hunan), Tongren (Guizhou), and Dujiangyan (Sichuan)—were randomly selected from eastern, central, and western economic zones as defined by the National Bureau of Statistics. These cities were chosen for their geographic, economic, and urbanization diversity. Next, two public junior middle schools were randomly selected from each city, totaling 12 schools, all standard public institutions to control for resource disparities. Finally, one class per grade (grades 7–9) was randomly chosen from each school for survey administration, with homeroom teachers assisting in questionnaire distribution.of 2200 questionnaires distributed, 1738 valid responses were retained after excluding invalid submissions (e.g., patterned or blank responses; see Fig. 1). The study protocol was approved by the Ethics Committee of Jishou University (Approval No. JSDX-2023-0034), and informed consent was obtained from all participants.

## Measurements

### Independent variable

Family economic status(FES). This variable is measured using a scale of household wealth adapted from (Currie et al., 2008) [20]. This self-reported measure assesses factors such as whether the family owns a car and if they have their own private bedroom. The scale ranges from 0 to 9 points, scored on a 5-point scale: ① represents ‘low level’ (0–1 points), ② represents ‘lower level’ (2–3 points), ③ represents ‘average level’ (4–5 points), ④ represents ‘higher level’ (6–7 points), and ⑤ represents ‘high level’ (8–9 points). This scale has high adaptability among Chinese adolescents [21]. In this study, the Cronbach’s α coefficient for this scale was 0.808.

Family structure(FS). This variable is scored on a 4-point scale, where ① represents ‘single-parent family (living only with father or mother)’, ② represents ‘blended family’, ③ represents ‘three-generation family (living with grandparents, parents)’, and ④ represents ‘nuclear family (living with parents)’. Since the family structure variable consists of only one item, it is not included in the internal consistency test.

Family sports atmosphere(FSA). This variable is primarily measured using a survey questionnaire designed by (Guo WF, 2024) [22], consisting of 5 items such as the frequency of family members engaging in sports activities together. The scale uses a 5-point scoring system, where ① represents ‘almost never’, ② represents ‘once or twice a week’, ③ represents ‘three to four times a week’, ④ represents ‘five to six times a week’, and ⑤ represents ‘seven or more times a week’. This scale has high reliability and validity among Chinese adolescents [23].In this study, the Cronbach’s α coefficient for this scale is 0.811.

Positive parenting style(PPS). This study employed the Chinese version of the Short Form of the EMBU (S-EMBU-C), which was translated and revised by (Jiang et al., 2010) [24]. The scale includes two subscales targeting fathers and mothers, each comprising 21 items and three dimensions: rejection (6 items), emotional warmth (7 items), and overprotection (8 items). To maintain measurement consistency, this study, based on existing research evidence [25, 26], used the emotional warmth dimension to represent parents’ positive parenting style. This dimension consists of seven items (items 2, 6, 9, 11, 13, 17, and 21) that assess Chinese parents’ care and affection for their children. Each item is scored on a 4-point scale, where ① represents ‘never,’ ② represents ‘occasionally,’ ③ represents ‘often,’ and ④ represents ‘always.’ The father’s and mother’s dimensions were combined into a single parental dimension in this study to measure overall family positive parenting style. A higher total score indicates a higher degree of positive parenting style adopted by parents, characterized by warmth, understanding, support, care, and parental involvement. The scale has high applicability in Chinese adolescent populations [25]. In this study, the Cronbach’s α coefficient of the scale was 0.861.

Parent-child relationship(PR). This variable refers to previous studies [27], including 4 positively worded items: ‘I feel happy interacting with my parents,’ ‘I share my inner secrets and personal feelings with my parents,’ ‘My parents help me solve problems when I encounter them,’ and ‘I do enjoyable activities with my parents,’ along with 1 negatively worded item: ‘My parents and I argue or blame each other.’ All five items are scored on a 5-point scale, with the negatively worded item reversed for scoring. A higher total score indicates a better parent-child relationship.This scale has high adaptability among Chinese adolescents [28]. In this study, the Cronbach’s α coefficient for this scale was 0.861.

### Result variable

Physical Activity (PA). This variable was assessed using the revised Physical Activity Rating Scale-3 (PARS-3) by (Dong BL, 2018) [29]. This scale primarily evaluates activity levels based on three aspects: intensity, duration, and frequency of exercise during leisure time. Exercise level is calculated using the formula ‘exercise volume = intensity × duration × frequency’ (with a maximum score of 100 and a minimum score of 0), where intensity and frequency are rated from 1 to 5, and duration is rated from 1 to 5, corresponding to scores of 1 to 5 and 0 to 4, respectively. Based on survey results, a 4-point scoring system was used, with ① rrepresents ‘sedentary activity’ (0 points), ② represents ‘low activity’ (1–19 points), ③ represents ‘moderate activity’ (20–42 points), and ④ represents ‘high activity’ (more than 43 points). This scale has high reliability and validity among Chinese adolescents [30]. In this study, the Cronbach’s α coefficient for this scale was 0.832.

### Data management and quality control

To ensure the authenticity and reliability of the data, this study conducted a pilot survey (*n* = 120) before the formal investigation to examine the validity and reliability of the scale in the target population. Cronbach’sαcoefficient was used to test the internal consistency reliability of the scale, with allαcoefficients > 0.70, indicating good reliability; exploratory factor analysis was performed on the items using principal component analysis combined with varimax rotation, where the factor loadings of all items were > 0.40, and the cumulative variance contribution rate met the requirements, verifying that the scale’s factor structure is basically consistent with the theoretical construct. It should be noted that the pilot survey data was only used for tool validation and not included in the final analysis. During the formal data collection and management, systematic quality control measures were implemented, such as training investigators, standardizing the questionnaire distribution process, and double-blind independent data entry. Analysis revealed that the missing rate of each variable ranged from 1.2% to 3.5%, and Little’s MCAR test (*P* > 0.05) supported that the data were missing at random (MAR). Five complete datasets were generated using multiple imputation to reduce bias from missing data and enhance the robustness of statistical inference [31]. Finally, data cleaning was performed in SPSS 25.0, excluding non-effective responses (such as patterned answers or questionnaires with > 20% missing items) and outliers, resulting in a high-quality dataset for analysis.

### Statistical analysis

Use SPSS 25.0 software for descriptive statistics and correlation analysis of the data. Count data were expressed as percentages (%) and Quantitative data were represented by ($\overline{x}\pm{s}$). To explore complex causal relationships between multiple condition configurations and outcomes, fsQCA 3.0 software was used for nonlinear statistical analysis. First, the raw data were transformed into 0–1 fuzzy set membership scores, with calibration anchors set at full membership (1), crossover point (0.5), and full non-membership (0). The raw data were calibrated using the direct method. Second, each condition was tested to determine if it was a necessary condition for the outcome, requiring consistency (Consistency) ≥ 0.9 and significant coverage (Coverage). Third, the calibrated data were converted into truth tables, with case frequency thresholds typically ≥ 1 and consistency thresholds ≥ 0.8. Logical remainders were simplified to generate configuration solutions, and logical operations produced parsimonious solutions (retaining only logical remainders), complex solutions (excluding logical remainders), and intermediate solutions (combining theory and counterfactual analysis). This study primarily reports intermediate solutions. Finally, the explanatory power of the configurations was assessed through consistency (≥ 0.75) and coverage (original coverage, unique coverage), and subset relationships among conditions were examined. The advantage of fsQCA is its ability to identify multiple concurrent causal paths, allowing asymmetric effects of conditions on outcomes (e.g., A→Y and ~ A→Y may coexist). It can handle both small samples (< 50 cases) and large samples (thousands of cases) and is applicable to various types of data (such as categorical variables, Likert scales, etc.) [32]. The significance level α = 0.05.

## Results

### Descriptive statistics and related analysis

This study included a total of 1,738 middle school students with a mean age of (13.49 ± 0.92) years. There were 980 boys (56.4%) and 758 girls (43.6%), with 8th-grade students comprising the largest proportion (54.1%). The sample was well-balanced in terms of school location (51.6% urban, 48.4% rural) and geographical region (36.5% Eastern, 35.4% Central, 28.1% Western). Regarding family background, nearly half (49.0%) of the students were from nuclear families, and 29.9% were from three-generation families. The vast majority (81.8%) had parents who were married. In terms of biological custody, 79.9% of students lived with both biological parents. Finally, 48.0% of the students were only children. See Table 1.

**Table 1:** Distribution of characteristics in the adolescent health study sample (*N* = 1,738)

Characteristic	Category	Value (*N*, %) or Mean ± SD
demographic characteristics		
Age (years)		13.49 ± 0.92
Gender	Boys	980 (56.4%)
	Girls	758 (43.6%)
Grade	Seventh grade	475 (27.3%)
	Eighth grade	940 (54.1%)
	Ninth grade	323 (18.6%)
School characteristics		
School location	City	897 (51.6%)
	Countryside	841 (48.4%)
Region	Eastern region	634 (36.5%)
	Central Region	615 (35.4%)
	Western region	489 (28.1%)
Family structure and background		
Number of children	Only child	835 (48.0%)
	Two children	728 (41.9%)
	Three or more	175 (10.1%)
Parental Marital Status	Married	1421 (81.8%)
	Divorced	237 (13.6%)
	Separated	42 (2.4%)
	Widowed	22 (1.3%)
	Other (e.g., Never married)	16 (0.9%)
Family Custody Type	Both biological parents	1389 (79.9%)
	Single biological parent	214 (12.3%)
	Stepparent/Blended family	152 (8.7%)
	Other (e.g., Grandparent, Foster)	21 (1.2%)
Family Structure Type	Nuclear family	852 (49.0%)
	Three-generation family	520 (29.9%)
	Single-parent family	214 (12.3%)
	Blended family	152 (8.7%)

Data are presented as mean ± standard deviation (Mean ± SD) for age and as number (percentage) for categorical variables

From the correlation matrix of variables (Table 2), it can be seen that family economic status, positive parenting styles, and family sports atmosphere are positively correlated with adolescent sports activities (*P* < 0.01). In addition, there are correlations among the independent variables (*P* < 0.01), such as between family structure and positive parenting styles. In other words, these variables are not mutually independent, so traditional linear research methods are insufficient to explain this issue.

**Table 2:** Correlation coefficient matrix between variables (*N* = 1,738)

variable	M	SD	1	2	3	4	5
1.Family economic status(FES)	4.03	0.57	1.00				
2.Family structure(FS)	2.85	0.92	0.13	1.00			
3.Positive parenting style(PPS)	3.87	0.75	0.57**	0.55**	1.00		
4.Parent-child relationship(PR)	4.21	0.59	0.69**	0.61**	0.17	1.00	
5.Family sports atmosphere(FSA)	3.48	0.54	0.52**	0.56**	0.53**	0.59**	1.00
6.Physical activity(PA)	4.23	0.52	0.76**	0.51**	0.58**	0.57**	0.72**

Correlation coefficients were calculated using Pearson product-moment correlation.1 = FES, 2 = FS, 3 = PPS, 4 = PR, 5 = FSA, 6 = PA

*M* Mean, *SD* Standard Deviation

** indicates *P* < 0.01 (two-tailed test)

### Variable calibration

To ensure the transparency and scientific rigor of the fuzzy-set calibration, the selection of anchors for full membership, crossover point, and full non-membership for each variable was based on a comprehensive consideration of the theoretical meaning of the variables, the distribution characteristics of the actual data, and empirical evidence from previous studies [33]. First, descriptive statistics and distribution checks (such as skewness and kurtosis) were performed for all continuous variables. Second, substantively meaningful calibration anchors were established based on theory (e.g., scale design) and the actual distribution of the sample data (e.g., the 25th percentile, median, and 75th percentile). The specific rationale and anchor values for each variable are detailed in Table 3. Subsequently, using these anchors, the original data were transformed into fuzzy-set membership scores ranging from 0 to 1 through the direct calibration method in fsQCA 3.0 software.

**Table 3:** Descriptive statistics and calibration anchor rationale for variables

Variable Name	Mean ± SD	Theoretical Range	Calibration Anchors (Full Membership / Crossover / Full Non-Membership)	Rationale for Calibration
Family Economic Status (FES)	4.03 ± 0.57	1–5	**5 / 3 / 1**	Based on the 5-point scale. Theoretically, a score of 5 represents a ‘high level’ and 1 represents a ‘low level’. The median score of 3 represents an ‘average level,’ serving as the natural theoretical threshold distinguishing high from low.
Family Structure (FS)	2.85 ± 0.92	1–4	**4 / 3 / 1**	Based on comprehensive considerations, the ‘family integrity’ dimension was established. Nuclear families (4) were calibrated as full members (1.0), representing the most favorable family structure for adolescents’ healthy development; single-parent families (1) and reconstituted families (2) were calibrated as non-members (0.0), representing non-intact families; extended families with three generations living together (3) were calibrated as crossover points (0.5), because their functions are similar to those of nuclear families but there are structural differences, serving as a fuzzy transition point between intact and non-intact families. Preliminary data analysis showed no significant difference in the achievement rate of moderate to vigorous physical activity among adolescents in nuclear families and extended families (χ²=0.35, p = 0.55), providing empirical support for this calibration.
Positive Parenting Styles (PPS)	3.87 ± 0.75	1–4	**4 / 3 / 1**	Based on the 4-point scale. A score of 4 represents ‘always’ experiencing emotional warmth, while 1 represents ‘never’. The median score of 3 (‘often’) is the key theoretical threshold distinguishing positive from negative parenting styles.
Parent-Child Relationship (PR)	4.21 ± 0.59	1–5	**5 / 3 / 1**	Based on the 5-point scale. A score of 5 indicates a ‘very good’ relationship, and 1 indicates a ‘very poor’ one. Referring to the data distribution, the 25th percentile was around 3.5, but to align with the theoretical midpoint and enhance discriminative power, a score of 3 was chosen as the crossover point.
Family Sports Atmosphere (FSA)	3.48 ± 0.54	1–5	**5 / 3 / 1**	Based on the 5-point scale. A score of 5 represents the ideal state of ‘7 or more times per week’ of joint activity, while 1 represents ‘almost never’. The median score of 3 (‘3–4 times a week’) is the theoretical and practical threshold distinguishing active from inactive family atmospheres.
Physical Activity (PA)	4.23 ± 0.52	1–4	**4 / 2 / 1**	Based on the 4-point scale. A score of 4 represents ‘high activity level,’ which is the desired outcome. A score of 1 represents ‘sedentary behavior’. The median score of 2 (‘low activity level’) is the key threshold for distinguishing whether basic activity levels are m**et.**

### Analysis of the necessity of a single condition

In fsQCA, if a condition is always present when the outcome occurs, it is considered a necessary condition for the outcome. Consistency is an important criterion for testing necessary conditions; when consistency exceeds 0.9, the condition is deemed a necessary condition for the outcome [33]. As shown in Table 4, the analysis of necessary conditions for adolescent physical activity using fsQCA software reveals that all antecedent conditions have a consistency below 0.9, indicating that none of the antecedent conditions alone constitute a necessary condition for adolescent physical activity behavior. This also means that the antecedent conditions cannot effectively explain adolescent physical activity behavior. Therefore, to interpret the mechanisms driving adolescent physical activity behavior, one must consider the synergistic effects of multiple conditions.”

**Table 4:** Analysis of the necessity of individual conditions

Variable	Consistency	Coverage
FES	0.83472	0.82641
~FES	0.44653	0.51093
FS	0.72341	0.65454
~FS	0.52204	0.70306
PPS	0.74341	0.65243
~PPS	0.51876	0.72137
PR	0.62754	0.63858
~PR	0.54835	0.71536
FSA	0.53941	0.75569
~FSA	0.61543	0.52857

“~” indicates “not”, and the same applies below

### Constructing truth tables

Generally, sample data should have a higher frequency threshold, retaining at least 75% of the sample size to reduce potential conflicting configurations. The minimum PRI consistency should be ≥ 0.75, with the smallest discrimination threshold being 0.75 and considering natural breakpoints for consistency [33, 34]. This study, combining previous research experience, sets the case frequency threshold at 5 (only considering condition combinations with a case count ≥ 5, which can retain about 85% of the sample size, ensuring configuration robustness and reducing random noise), and the consistency threshold at 0.8 [35]. Rows that meet the frequency and consistency thresholds are marked as 1 (high physical activity) if the result is 1, and as 0 (non-high physical activity) if the result is 0. Conflicting rows are handled by checking the original consistency and PRI consistency; if the PRI consistency is below 0.75, it is considered ambiguous and not retained; if the PRI consistency is high but there are still conflicts, individual characteristics are reviewed. In this study, all retained rows have a PRI consistency above 0.75, with clear subset relationships and negligible conflicts. Ultimately, the truth table retains 10 condition combinations, see Table 5, followed by simplifying the logical remainder to generate the configuration solution.

**Table 5:** Complete list of retained rows from the truth table (Frequency ≥ 5)

FES	FS	PPS	PR	FSA	Number	PA	Raw consist.	PRI consist.	SYM consist.
1	1	0	0	0	187	1	0.995814	0.953617	0.964518
1	1	0	1	0	165	1	0.971317	0.903035	0.903035
1	0	1	0	1	152	1	0.936874	0.894315	0.897658
1	1	1	0	1	138	1	0.917649	0.853402	0.853403
1	1	1	1	1	125	1	0.871867	0.798757	0.798759
1	0	0	1	0	98	1	0.972143	0.767354	0.767352
1	0	1	1	1	85	0	0.909151	0.746021	0.746023
1	1	1	0	0	72	0	0.916742	0.706843	0.706846
1	1	1	1	0	63	0	0.874832	0.606145	0.606146
0	0	1	1	0	51	0	0.645225	0.126878	0.126878
…	…	…	…	…	…	…	…	…	…
(Other rows omitted for illustration)									

Rows are ordered by raw consistency for outcome (PA=1) in descending order. Only rows with frequency ≥ 5 are shown

*FES* Family economic status, *FS* Family structure, *PPS* Positive Parenting styles, *PR* Parent-child relationship; *FSA* Family sports atmosphere, *PA* Physical activity, *Number* Number of cases, *Raw consist* Raw consistency, *PRI consist* Proportional Reduction in Inconsistency consistency, *SYM consist* Symmetric consistency

### Path configuration analysis

In this study, solid circles indicate the presence of conditions, crossed circles indicate the absence of conditions, and blank spaces indicate that conditions may either be present or absent. Large circles represent core conditions (conditions present in both simplified solutions and intermediate solutions), while small circles represent auxiliary conditions (conditions present only in intermediate solutions). Table 6 shows four paths used to explain the driving factors behind adolescent physical activity behavior, with each column representing a possible condition configuration. The consistency of the overall solution is 0.93, indicating that among adolescents meeting these four condition configurations, 93% participate in physical activity behavior. The coverage rate of the overall solution is 0.82, indicating that these four condition configurations can explain 82% of adolescent physical activity behavior. Both the consistency and coverage rates exceed the critical value of 0.75, suggesting that the analysis results are valid.

**Table 6:** Results of configurational analysis for adolescent physical activity

Independent variable	Path A	Path B	Path C	Path D
Family economic status(FES)	**λ**	**l**	**l**	**⮾**
Family structure(FS)	**⮾**			**l**
Positive parenting styles(PPS)		**l**	**⮾**	**l**
Parent-child relationship(PR)	**⮾**		**l**	
Family sports atmosphere(FSA)	**l**		**⮾**	**l**
Raw coverage	0.435786	0.409458	0.430785	0.461426
Uunique overage	0.042147	0.048764	0.043659	0.041351
Consistency	0.973215	0.965827	0.981432	0.926741
Solution consistency	0.936281			
Solution coverage	0.820743			

●: Core condition present, this condition appears in both the minimal solution and the intermediate solution.

l༚Core condition absent, the absence of this condition occurs in both the minimal solution and the intermediate solution

⮾༚Peripheral condition present, this condition only appears in the intermediate solution

⮾༚Peripheral condition absent, the absence of this condition only appears in the intermediate solution

Blank spaces༚ Irrelevant condition, this condition may or may not exist in this path, and its status does not affect the result

## Discussion

This study found both consistencies and important extensions compared to previous regression studies. For example, a meta-analysis showed that single factors such as parental support and behavioral modeling had only stable but weak to moderate positive correlations with adolescent physical activity [36]. Similarly, another study also found weak correlations between parental and children’s activity levels (average *r* = 0.13) [37]. These findings are basically consistent with our research results. The necessity analysis of this study indicated that single family factors are not necessary conditions for adolescent physical activity, explaining the limited explanatory power of individual variables. However, configurational analysis further revealed that family factors need complex synergistic and alternative mechanisms to function, with multiple equivalent paths driving adolescents’ high-level physical activity (i.e., ‘different roads lead to Rome’). This finding goes beyond the limitations of regression studies, emphasizing the multidimensionality and integrality of family influence, and provides new theoretical basis for developing precise intervention strategies for different family backgrounds.

### Equivalent paths driving adolescent sports activities

*Path (A)*: Family Economic Status (FES) * ~Family Structure (FS) * ~Parent-Child Relationship (PR) * Family Sports Atmosphere (FSA) (The symbol ‘*’ represents ‘with’, as noted below). The original coverage of this path is 43.58%, and the unique coverage is 4.21%. This indicates that even under structural disadvantages such as single-parent or reconstituted families (~ FS) and less harmonious parent-child relationships (~ PR), a superior family economic status (FES) combined with a strong family sports atmosphere (FSA) can still effectively drive adolescents’ sports activities. The core mechanism of this path may originate from observational learning and imitation in social cognitive theory [5]. A high family economic status provides a resource buffer for purchasing sports equipment and accessing paid sports facilities [9, 12]. Meanwhile, a strong family sports atmosphere (FSA) provides a continuous behavioral model, allowing adolescents to observe and imitate their family members’ sports activities, thereby offsetting the potential negative impacts of incomplete family structure and tense parent-child relationships [17, 22–24].

*Path (B)*: Family Economic Status (FES) * Positive Parenting Style (PPS). This path can explain about 40.95% of the sample, with a unique coverage rate of 4.88%. This indicates that when structural and process factors such as family type and parent-child sports are absent, the combination of high family economic status (FES) and emotionally warm parenting style (PS) can stimulate adolescents’ sports activity behavior. The core mechanism box of this path may highlight the direct interaction within the microsystem in the social ecological model [37, 38]. An emotionally warm parenting style (PS) effectively enhances adolescents’ self-efficacy through providing autonomy support and psychological security [10, 28], which is a key personal trait for promoting behavioral change. Family economic capital (FES) provides instrumental support for realizing this behavior [12, 36]. Together, the two can stimulate adolescents’ intrinsic motivation to participate in sports activities.

*Path (C)*: Family Economic Status (FES) * ~Positive Parenting Style (PPS) * Parent-Child Relationship (PR) * ~Family Sports Atmosphere (FSA). This path can explain about 43.08% of the sample, with a unique coverage rate of 4.37%. This path shows that during the process where parents adopt an overprotective parenting style (~ PS) and the family lacks a sports atmosphere (~ FSA), high family economic status (FES) and harmonious parent-child relationship (PR) can serve as an alternative driving force. The core mechanism box of this path may reflect the compensatory effect of protective factors in the social ecological model [39, 40]. Negative parenting styles (such as overprotection) and the absence of a sports atmosphere are risk factors, but a harmonious parent-child relationship (PR), as emotional support and relational assets [41, 42], can buffer their negative impacts. Communication and trust in high-quality parent-child relationships help adolescents understand and accept the concerns behind parents’ overprotection, converting it into motivation for safe participation in activities, while economic resources (FES) ensure the feasibility of these activities [43–45]. Existing literature indicates that overprotective parenting styles are associated with low physical activity [10], but this study found that under high-quality parent-child relationships, their negative impacts can be mitigated.

*Path (D)*: ~Family Economic Status (FES) * Family Structure (FS) * Positive Parenting Style (PPS) * ~Family Sports Atmosphere (FSA). This path can explain about 46.14% of the sample, with a unique coverage rate of 4.14%. This indicates that under the constraints of poor family economic status (~ FES) and lack of sports atmosphere (~ FSA), a sound nuclear family structure (FS) and emotionally warm parenting style (PS) can synergistically compensate to drive adolescents’ sports activities. The core mechanism box of this path may reflect the substitution effect of family capital [9, 12]. According to the Family Investment Model [46], the lack of economic capital (FES) can be compensated by strong human capital (such as parents’ scientific parenting knowledge - PS) and social capital (such as stable family structure - FS). Intact two-parent families (FS) can provide more sufficient emotional attention and time investment, and scientific parenting styles (PS) can convey healthy values and behavioral norms to adolescents, shaping their attitude toward sports (Attitude) and subjective norms (Subjective Norm) [8, 47–49], overcoming economic and physical environmental restrictions.

### Substitution effects driving adolescent physical activity behavior

#### Substitution effects between structural factors

 Through comparative analysis of configuration A and configuration D, it is evident that when the family sports atmosphere is strong, there is a substitution effect between family economic status and family structure. In other words, for adolescents in families with a strong sports atmosphere: if their family structure undergoes unfavorable changes, having a higher family economic status can drive participation in physical activities; if the family’s economic conditions are low, a healthy family structure can also motivate adolescents to engage in physical activities. Therefore, for adolescents in families with a sports atmosphere, there is a mutual substitution effect between family economic status and family type.

#### Substitution effects among process factors

Comparing configuration B and configuration C, in families where family type is absent but economic status is high, parenting styles and parent-child relationships exhibit a mutual substitution relationship. In this context, emotionally warm parenting can drive adolescents’ participation in physical activities, while harmonious parent-child relationships can also motivate adolescents to engage in physical activities. In other words, when family economic status is high, there is a lack of a family sports atmosphere, and parenting styles are not emotionally warm, there exists a mutual substitution effect between the parent-child relationship and parenting style within the process factors.

#### The substitutive effect between structural factors and process factors

In configurations A and B, it is observed that for teenagers with a strong family sports atmosphere, the combination of family economic status and parent-child relationships substitutes the combination of family type and parenting styles in driving physical activity behavior among adolescents. That is, for teenagers with a weak family sports atmosphere, either high family economic status combined with harmonious parent-child relationships, or a healthy and harmonious family type combined with emotionally warm parenting styles, can both drive the participation level in physical activities among teenagers. Therefore, for teenagers lacking a family sports atmosphere, the combination of process factors such as parent-child relationships and structural factors like family economic status substitutes the combination of process factors like parenting styles and structural factors like family type.

### Strengths and limitations

This study is the first to apply fuzzy-set qualitative comparative analysis (fsQCA) to the field of adolescent health behavior research, breaking through the limitations of traditional regression analysis and single-factor linear thinking. This method can effectively capture complex non-linear interactions and synergistic pathways among family factors, revealing multiple \“equivalent paths\“ and \“substitution effects\“ driving adolescents to achieve high levels of physical activity, thereby providing a new perspective for the implementation of intervention strategies at the family level. Although this study has certain advantages, it also has some limitations: First, all variables in this study were measured using self-report methods, which are susceptible to biases such as social desirability, inaccurate self-reflection, and limited self-awareness [50, 51]. Although the fsQCA method is relatively robust to random measurement errors, future studies should adopt a multi-method assessment strategy, such as combining device measurements (accelerometers) and reports from multiple informants (parents), to improve data validity. Second, the cross-sectional research design of this study limits causal inference and temporal precedence, which may affect the interpretation of mediating and moderating effects [52, 53]. The causal relationship between family environment and adolescent physical activity may be bidirectional; therefore, future studies should adopt a longitudinal tracking design to verify the causal direction of configurational relationships. Third, the generalizability of the research findings may be constrained by cultural background. Research results from one cultural context may not be applicable to others due to differences in norms, values, and ecological conditions [54, 55]. The patterns derived from this study originate from a Chinese sample, where unique family and cultural norms may have shaped the relationships between variables. Caution is needed when generalizing the conclusions to other cultural backgrounds, and future cross-cultural comparative studies can be conducted to explore commonalities and specificities across different cultural contexts. Finally, this study primarily focuses on core determinants within the family system but did not include external environmental factors such as peer influence and school sports policies. Although the family is the core microsystem, external factors may influence family effects. Future research can adopt an integrated ecological perspective to construct a holistic model spanning families, schools, and communities.

## Conclusions

The family economic status, family structure and process factors such as parents’ parenting styles, parent-child relationships, and family sports atmosphere do not constitute the necessary conditions for adolescents’ physical activity behaviors. The driving of adolescents’ physical activity behaviors requires the collaborative effect of structural factors and process factors. Using the configuration perspective and QCA method, four equivalent paths driving adolescents’ physical activity behaviors were identified. There are substitution effects among structural factors, process factors, and between the two. That is, when certain or some structural factors or process factors are absent, other structural factors, process factors, or combinations of factors can drive adolescents’ physical activity behaviors. This study goes beyond linear thinking that focuses only on the net effect of individual factors, emphasizing the multidimensionality and wholeness of family system influences, and provides a new theoretical basis for developing interventions tailored to families with different backgrounds.

## Data Availability

The datasets generated and/or analysed during the current study are not publicly available but are available from the corresponding author on reasonable request.

## Abbreviations

FES: Family Economic Status
FS: Family Structure
PS: Parenting Styles
PPS: Positive Parenting Styles
PR: Parent-child Relationship
FSA: Family Sports Atmosphere
PA: Physical Activity
